# A High Starch Diet Alters the Composition of the Intestinal Microbiota of Largemouth Bass *Micropterus salmoides*, Which May Be Associated With the Development of Enteritis

**DOI:** 10.3389/fmicb.2021.696588

**Published:** 2021-07-08

**Authors:** Xiaoli Huang, Liang Zhong, Qin Kang, Sha Liu, Yang Feng, Yi Geng, Defang Chen, Yangping Ou, Shiyong Yang, Lizi Yin, Wei Luo

**Affiliations:** ^1^Department of Aquaculture, College of Animal Science & Technology, Sichuan Agricultural University, Chengdu, China; ^2^Department of Basic Veterinary, College of Veterinary Medicine, Sichuan Agricultural University, Chengdu, China

**Keywords:** high starch diet, intestinal microbiota, enteritis, intestinal diseases, *Micropterus salmoides*

## Abstract

Starch is an inexpensive feed ingredient that has been widely used in fish feed. However, starch utilization by carnivorous fish is limited and excess starch is detrimental to the health of the organism. High starch diets often lead to liver damage, but the effects on the intestine are often overlooked. Therefore, in this study, two isonitrogenous and isolipidic semi-pure diets (NC: 0% α-starch, HC: 22% α-starch) were formulated and fed to largemouth bass (*Micropterus salmoides)* for 45 days. The effects of the high starch diet on the intestine of largemouth bass were comprehensively investigated by intestinal microbiota, histopathology, ultrastructural pathology, and enzymology analyses. Feeding the HC diet did not affect the growth of largemouth bass during the experimental period. However, the high starch diet led to a reduction in the diversity and abundance of intestinal microbiota in largemouth bass, with a significant increase in the abundance of harmful bacteria (*Aeromonas*) and a decrease in the abundance of beneficial bacteria (*Clostridium*, *Lactobacillus*, and *Bifidobacterium*). Feeding the HC diet caused the development of enteritis, with goblet cell hyperplasia, epithelial necrosis and detachment and inflammatory cell infiltration, and leading to enlarged apical openings and mitochondrial damage in goblet cells. Long-term feeding of the HC diet inhibited intestinal α-amylase activity. changes in the intestinal microbiota, such as an increase in *Aeromonas* and a decrease in *Clostridium*, *Lactobacillus*, and *Bifidobacterium*, may be closely related to the development of enteritis. Therefore, adding these beneficial bacteria as probiotics may be an effective way to prevent damage to the intestine of largemouth bass from a high carbohydrate diet. Our results suggest reducing the amount of starch added to the largemouth bass diets. This study provides a reference for protecting the largemouth bass gut during modern intensive culture.

## Introduction

Starch is a carbohydrate and is a source of nutrition for most animals. Due to its low cost, starch is widely used in various fish feeds. It has been reported that adding the appropriate amount of starch to the diet stimulates protein-sparing action (Li et al., 2020), improves feed adhesion and facilitates feed production (Zhang et al., 2020c), reduces environmental pollution (Asaduzzaman et al., 2010), and improves fish growth performance (Zhou et al., 2015). However, fish live in water where it is almost impossible to ingest plant starch; therefore, fish have very limited digestion, absorption, and metabolism of starch, particularly carnivorous fish (Stone, 2003). Previous studies have reported that long-term consumption of high starch feed by carnivorous fish leads to the accumulation of glycogen and fat in the liver (Zhang Y.M. et al., 2020) and to hyperglycemia (Kamalam et al., 2017), which can seriously affect the growth of farmed fish and even lead to death.

The intestines are one of the most important organs of aquatic animals, and they play an important role in a variety of physiological functions such as digestion and absorption of nutrients, and endocrine and immune activities (Banić et al., 2018). A large number of intestinal microbiota inhabit the intestines of animals, which affects health and nutrition in a variety of ways, such as enhancing metabolic capacity of the host, protecting the host from pathogens, and regulating gastrointestinal development (Bäckhed et al., 2005). A balanced intestinal microbiota is a prerequisite for normal physiological functioning. However, the intestinal microbiota is susceptible to changes in the external environment, of which the composition of the diet is one of the most important factors (Salonen and de Vos, 2014). The abundance and diversity of intestinal microbiota decreased significantly in the rice field eel (*Monopterus albus*) fed a high-fat diet (Peng et al., 2019). Falcinelli et al. (2017) reported that zebrafish intestinal microbiota is altered when the fish are fed a high-fat diet. Similar to a high-fat diet, high starch intake leads to disturbances in the intestinal microbiota of juvenile golden pompano (*Trachinotus ovatus*) and increases the abundance of harmful bacteria (Zhao et al., 2020). Therefore, a balanced intestinal microbiota is essential for the body, and when this balance is disturbed, it can lead to a variety of intestinal diseases, such as inflammatory bowel disease (IBD; Manichanh et al., 2012) or Cohn’s disease (Li et al., 2012) in mammals, and enteritis in grass carp (*Ctenopharyngodon idellus*) (Tuan et al., 2018) and sturgeon (Huang et al., 2020).

The largemouth bass (*Micropterus salmoides*) is a typical carnivore that is widely farmed around the world as a commercially valuable fish, with an annual production of more than 470,000 tons in China (Fisheries and Fisheries Administration, 2020). However, largemouth bass do not use starch efficiently, and excessive starch intake damages normal organ structure and function. Adding 25% carbohydrate to the diet results in a significant decrease in survival and a significant increase in blood glucose levels and liver vacuolation in largemouth bass (Amoah et al., 2008). It has also been reported that adding 20% starch restricts growth, and enhances oxidative damage in the liver and the accumulation of liver glycogen in largemouth bass (Lin et al., 2018). Zhang et al. (2020b) reported that adding only 15% starch results in significant whitening of the liver, a significant increase in hepatic vacuolization, and disturbances in hepatic glucose metabolism. However, the effects of high starch on the intestine are often overlooked. Therefore, this study aimed to systematically investigate the effects of starch intake on the intestine of largemouth bass. The results of this study are intended to provide a reference for protecting the intestines of cultured largemouth bass.

## Materials and Methods

### Experimental Diets

Two isonitrogenous (49% crude protein) and isolipidic (9% crude lipid) semi-purified diets were formulated to contain different levels of α-starch, such as the NC (0% α-cassava starch) and HC (22% α-cassava starch) diets, respectively (Table 1). Fish meal, casein, and soybean protein concentrate were used as the protein sources, and soybean oil and soybean lecithin were used as the lipid sources. All dry ingredients were crushed and sifted through 280 μm mesh, weighed according to the ratio and mixed manually for 10 min. The mixed ingredients were transferred to a pelletizer and processed into 2 mm diameter pellets. All diets were air-dried at room temperature (23–30°C) and stored at −20°C until use.

**TABLE 1:** Formulation and proximate chemical composition of the trial diets.

**Ingredients**	**Starch level in diets (g.kg^–1^)**	
	**NC**	**HC**
Fish meal^a^	490	490
Casein^a^	130	130
Soybean protein concentrate^a^	60	60
Soybean oil^a^	30	30
Soybean lecithin^a^	20	20
Yeast extract^a^	8	8
Ca(H_2_PO_4_)_2_^a^	10	10
Choline chloride^a^	3	3
Vitamin mixture^b^	8	8
Mineral mixture^c^	5	5
Carboxymethyl cellulose^*a*^	15	15
Lysine^a^	1	1
α-Cassava starch^a^	220	0
Zeolite powder^a^	0	220
Proximate compositions (g. kg^–1^, dry matter)		
Crude protein	490	491
Crude lipid	82	91
Ash	301	103
Starch	13	224

*^a^Supplied by Chengdu Sanwang Feed Ltd (Chengdu, China). ^b^Vitamin Premix (mg kg^–1^ diet): vitamin A, 32.00; vitamin D3, 16.00; vitamin E, 351.83; vitamin K3, 30.03; vitamin C, 3288.80; vitamin B1, 19.77; vitamin B2, 60.00; vitamin B6, 36.43; vitamin B12, 24.00; niacinamide, 80.80; calcium pantothenate, 75.10; folic acid, 6.73; inositol, 329.90; biotin, 32.00; L-carnitine, 102.03. ^c^Mineral mix (mg kg^–1^ diet): FeSO_4_ (Fe), 70.33; MgSO_4_ (Mg), 351.33; CuSO_4_ (Cu), 8.00; ZnSO_4_ (Zn), 99.70; MnSO_4_ (Mn), 19.50; CoCl_2_ (Co), 19.37; Ca (IO_3_)_2_ (I), 50.17; Na_2_SeO_3_ (Se), 4.00.*

### Feeding Trial and Experimental Conditions

Eighty healthy juvenile largemouth bass (initial length 9.23 ± 0.46 cm, initial weight 7.20 ± 1.15 g) were obtained from a commercial farm in Chengdu, Sichuan, China. The fish were acclimated and fed the NC diet for 2 weeks in plastic tanks. At the start of the experiment, the fish were fasted for 24 h and grouped after anesthesia with MS-222. The 80 fish were randomly distributed into two groups, containing a control group (fed the NC, 0% starch) and the experimental group (fed the HC, 22% starch). The dietary treatments were randomly assigned to three replicates. The daily diet was fed at 8% of body weight, the amount of which was divided into two parts on average and fed daily at 9:00 and 18:00 for 45 days. The daily amount of the diet offered to the fish was adjusted every 15 days by weighing the total weight of the fish in each tank.

During the experiment, the water was changed twice daily with advanced aeration. Water temperature was 24.9 ± 1.0°C, dissolved oxygen was >6 mg L^–1^, pH was 7–8, and ammonia-nitrogen was almost zero. The photoperiod was 12L:12D, with lights on from 8:00 to 20:00. Fish mortality was observed and recorded every day.

All animal handling procedures were approved by the Animal Care and Use Committee of Sichuan Agricultural University, following the guidelines for animal experiments of Sichuan Agricultural University, under permit number DY-2019202033.

### Chemical Analysis

The chemical composition analyses of the diets were conducted by standard methods (AOAC, 2005). Crude protein was determined using the Kjeldahl method (N × 6.25) (Kjeltec 2,300, FOSS, Hilleroed, Denmark). Crude lipid was determined by petroleum ether extraction (without acid hydrolysis) using Soxtec (Soxtec 2,055, FOSS, Denmark). Ash was determined by combusting at 550°C to constant weight in a muffle furnace (Shenyang Energy-saving Electric Furnace Factory, Shenyang, China). Starch content was analyzed by spectrophotometry (spectropolarimeter CP225D, Sartorius, Goettingen, Germany). Intestinal trypsin, α-amylase, lipase, and total protein were assayed using commercial kits (Nanjing Jiancheng Bioengineering Institute, Nanjing, China).

### Sample Collection

The fish were sampled 30 and 45 days after the feeding trial began. All fish were anesthetized with MS-222 before sampling and were weighed and measured for body length and intestinal length. The intestinal length was measured from the stomach-intestine junction to the posterior end of the intestine. Five fish were used for histopathological and ultrastructural pathological observations and ten fish were used to determine intestinal digestive enzyme activity during each of the two sampling periods. However, an additional nine fish were sampled at 45 days for high-throughput sequencing of the intestinal microbiota 16s rRNA of sequences.

### Histopathological Examination

The five fish sampled after 30 and 45 days were fixed in 10% neutral buffered formalin. After 2 days of fixation, the fish were trimmed into cassettes, dehydrated through graded ethanol solutions, cleared in xylene, and embedded in paraffin wax. Sections of 4 μm were prepared and mounted on slides for hematoxylin and eosin (H&E) and Alcian blue-periodic acid Schiff (AB-PAS) staining, respectively. The slides were examined under an optical microscope after staining. The number of goblet cells was measured in 6–8 well-oriented villi.

The degree of intestinal cell hyperplasia, necrosis and detachment of epithelial cells, inflammatory cell infiltration, and necrosis of the lamina propria was scored with reference to the modified grade scoring system established by Baums et al. (2013). Histological changes were assessed using a score ranging from 1 to 7, depending on the extent of the lesion: (1) unchanged; (3) mild; (5) moderate; and (7) severe.

### Electron Microscopy

Fresh intestinal tissue was placed in fixative (2.5% glutaraldehyde in pH 7.4 cacodylate buffer). The intestinal tissues were washed three times in PBS and post-fixed in 1% osmium tetroxide. The samples were dehydrated through ascending concentrations of alcohol and post-embedded in Araldite. Cross-oriented ultra-thin sections were cut and stained with uranyl acetate and lead citrate. Images were acquired on a HITACHI HT7700 transmission electron microscope (Tokyo, Japan).

### High-Throughput Sequencing of the Intestinal Microbiota 16s rRNA

#### Collection of Intestinal Contents

At the end of the trial, the intestinal contents of nine largemouth bass from each treatment were sampled. The ventral surface of the fish was opened to expose the peritoneal cavity under sterile conditions. Thereafter, the entire intestine was excised, and rinsed several times in 0.65% sterile saline. The intestinal contents were collected in a sterile Eppendorf tube. The samples were kept on ice for less than 2 h and then stored at −80°C, pending analysis.

#### DNA Extraction and Purification

Genomic DNA was extracted from the intestinal contents using a bacterial DNA isolation kit (Foregene Company, Limited, China), according to the manufacturer’s instructions. After extraction, the genomic DNA was detected by 1% agarose gel electrophoresis. Samples were used for polymerase chain reaction (PCR) amplification with the forward primer (338F: 5′- ACTCCTACGGGAGGCAGCAG-3′) and the reverse primer (806R: 5′- GGACTACHVGGGTWTCTAAT-3′). The PCR product was detected by 2% agarose gel electrophoresis and purified with the AxyPrep DNA Gel Extraction Kit (Axygen, Corning, NY, United States), quantified using the QuantiFluorTM-ST Blue Fluorescence System (Promega, Beijing, China), and subjected to next-generation sequencing.

#### Sequencing, Processing and Analysis

Sequencing of the 16S rDNA was performed on an Illumina Miseq PE300 platform (Illumina, San Diego, CA, United States) by Meiji Bioinformatics Technology Company, Limited (Shanghai, China). The library was constructed for the V3–V4 amplicons, and paired-end (PE) sequencing was performed on the MiSeq system. The sequencing data were uploaded to the Sequence Read Archive at the National Center for Biotechnology Information (Accession number PRJNA730220). Based on the overlapping relationship between PE reads, pairs of reads were merged into a sequence using Flash software. Raw fastq files were demultiplexed, quality-filtered with the following criteria: (i) the 300 bp reads were truncated at any site receiving an average quality score <20 in a 50 bp sliding window, discarding the truncated reads <50 bp, (ii) splicing pairs of reads into a sequence based on the overlapping relationship between PE reads, assembling only sequences with an overlap of more than 10 bp, (iii) the maximum mismatch ratio allowed in the overlap region of a spliced sequence was 0.2, after removing non-conforming sequences, and (iv) exact barcode matching and two nucleotide mismatches in primer matching were removed (Zhou et al., 2016). The remaining sequences were clustered into operational taxonomic units (OTUs) with a similarity cutoff of 97%, and the OTU taxonomic analysis was performed using the Usearch pipeline^1^ (Zhang Y.M. et al., 2020). Each OTU was compared with the 16s rRNA database (Silva), using a BLAST analysis to obtain species classification information. A species composition analysis was conducted using Circos software. Species with a relative abundance rate of <0.01 in all samples were classified as “others.” The Shannon and Simpson indexes were used to assess community diversity, and the Ace and Chao indexes were used to assess community richness. The Venn diagram was prepared and the alpha diversity analysis was performed with Mothur^2^ (Zhang et al., 2020c). An effect size analysis (LEfSe) was performed to characterize the microbial differences between the two groups, such as a linear discriminant analysis (LDA). The non-parametric factorial Kruskal–Wallis rank-sum test was used to detect significant differences between assigned taxa, and the LDA was used to quantify the effect size of each feature with an alpha value of <0.05 (Zhang et al., 2020c).

### Statistical Analysis

All data are expressed as mean ± standard deviation. The experimental data were first tested for homogeneity of variance, using one-way analysis of variance. Non-normal data were subjected to the non-parametric Games-Howell test. Statistical analyses were performed using IBM SPSS 20.0 software (IBM Corp., Armonk, NY, United States), and a *P*-value < 0.05 was considered significant.

## Results

### The Effects of High Dietary Starch on Growth Performance of Largemouth Bass

**FIGURE 1:**
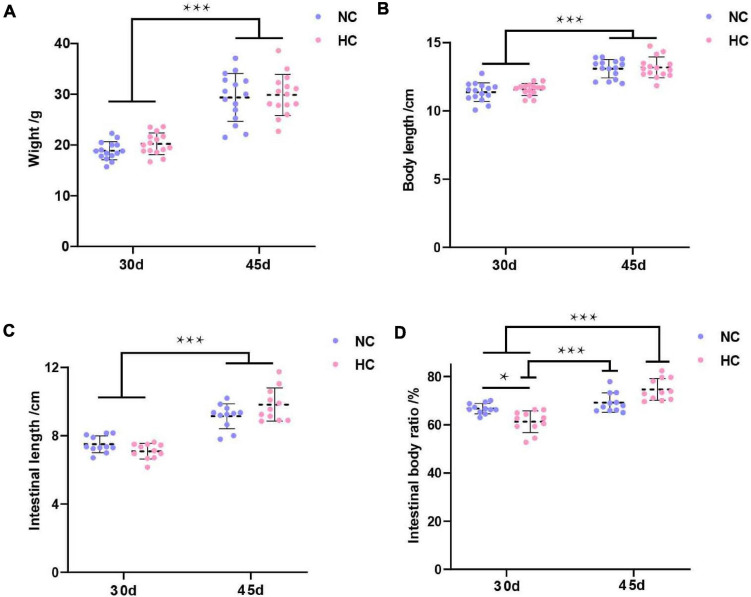
Growth performance of largemouth bass **(A–D)** Weight, body length, intestinal length, and intestinal body ratio of largemouth bass after 30 and 45 days, respectively (*P < 0.05; ***P < 0.001. NC: 0% α-starch diet, HC: 22% α-starch diet).

The growth performance of largemouth bass during the experiment is shown in Figure 1. The results showed no significant differences in weight, body length, or intestinal length between the two treatment groups after 30 and 45 days, respectively, but these indicators were significantly higher on day 45 than on day 30 (*P* < 0.001) (Figures 1A–C). Interestingly, the intestinal body ratio of fish fed the NC diet was significantly higher on day 30 than that of fish fed the HC (*P* < 0.05), but an opposite trend was detected at 45 days (Figure 1D).

### High Starch Feed Alters the Intestinal Microbiota Composition of Largemouth Bass

The effect of a high starch diet on the intestinal microbiota of largemouth bass was examined by 16S high-throughput sequencing. A total of 279,431 high-quality valid sequences were obtained with an average sequence length of 417 bp (Supplementary Table 1). In total, 650 OTUs were identified in the two groups of six samples, with 97% similarity. These OTUs belonged to 425 genera, 251 families, 159 orders, 65 classes, and 29 phyla. The rarefaction curve of all samples was flat (Supplementary Figure 1), indicating that all samples were sequenced.

**FIGURE 2:**
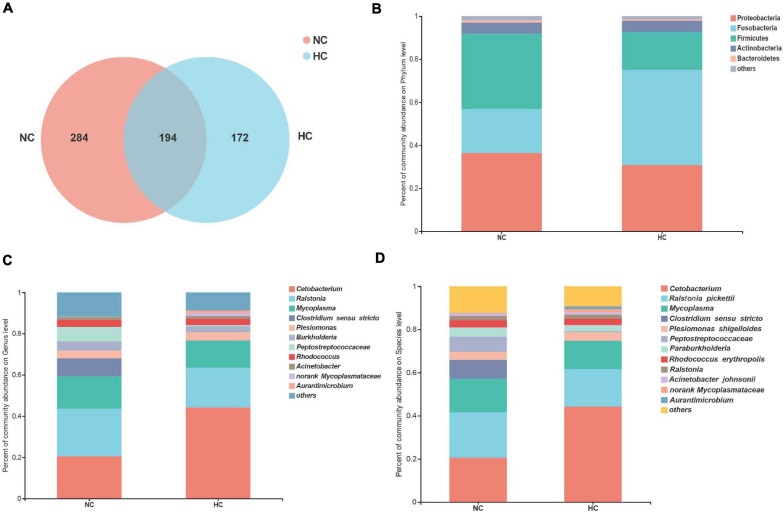
Analysis of the composition of the intestinal microbiota of largemouth bass fed the NC and HC diets. **(A)** Venn diagram of unique and shared OTUs. **(B–D)** intestinal microbiota composition at the phylum, genus, and species levels, respectively (NC: 0% α-starch diet, HC: 22% α-starch diet).

The results of the alpha diversity analysis showed that fish fed the NC diet had higher diversity and richness values of the intestinal microbiota than the fish fed the HC diet (Supplementary Table 2). A total of 194 OTUs were shared by fish fed both levels of starch, and fish fed the NC diet contained 284 unique OTUs, and those fed the HC diet only 172 OTUs (Figure 2A), suggesting that the high starch diets led to reduced OTU counts in the intestinal microbiota of largemouth bass. These results indicate that the high starch diet reduced the diversity and richness of the largemouth bass intestinal microbiota.

Proteobacteria, Firmicutes, Fusobacteria, Actinobacteria, and Bacteroidetes were identified as the dominant phyla in fish fed both diets, but their relative abundance differed between the two groups. The abundance of Fusobacteria increased in fish fed the HC diet, whereas the abundance of Proteobacteria and Firmicutes decreased in fish fed the HC diet compared to the fish fed the NC diet (Figure 2B). *Ralstonia*, *Cetobacterium*, *Mycoplasma*, *Clostridium sensu* stricto, and Peptostreptococcaceae were the top five dominant genera in fish fed the NC diet, while *Cetobacterium*, *Ralstonia*, *Mycoplasma*, *Plesiomonas*, and *Rhodococcus* were dominant in fish fed the HC diet (Figure 2C). Four species were identified in both groups, namely, *Ralstonia pickettii*, *Plesiomonas shigelloides*, *Rhodococcus erythropolis*, and *Acinetobacter johnsonii* (Figure 2D). These results suggest that the intestinal microbiota of largemouth bass was affected by the high starch diet.

### The High Starch Diets Reduce the Relative Abundance of Beneficial Intestinal Bacteria in Largemouth Bass

**FIGURE 3:**
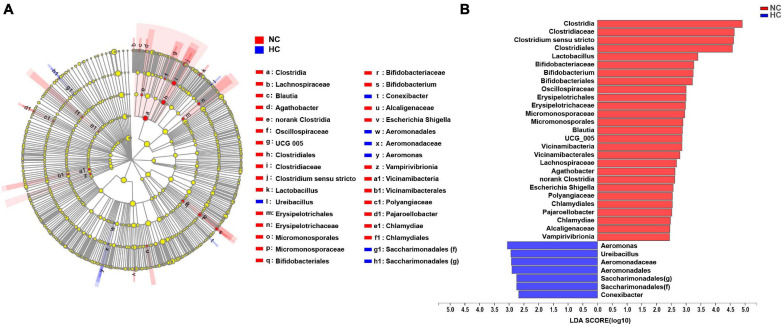
LEfSe analysis of the differences in the intestinal microbiota of largemouth bass fed two diets **(A)** Taxonomic representation of differences in the intestinal microbiota of largemouth bass treated with the NC or HC diets. The concentric circles from the inside out represent the different taxonomic classes (phylum to genus). The different colored nodes indicate differences in intestinal microbiota (red represents a significantly higher abundance of intestinal microbiota in the NC treatment than in the HC treatment, while blue represents the opposite, and yellow indicates no significant difference). The size of each node indicates the abundance of intestinal microbiota. **(B)** Histogram of linear discriminant analysis (LDA) scores for differential abundance of the intestinal microbiota.

A LEfSe LDA analysis was performed to further analyze the differences in intestinal microbiota composition between fish fed the NC and the HC diets. The results revealed significant differences in the intestinal microbiota of largemouth bass fed the NC and the HC diets (Figure 3). The relative abundance of order *Aeromonadales*, family Aeromonadaceae, Saccharimonadales (f), genus *Aeromonas*, *Ureibacillus*, *Saccharimonadales* (g), *Conexibacter* in the intestinal microbiota of fish fed the HC diet increased significantly compared to fish fed the NC diet; however, the relative abundance of class Clostridia, Vicinamibacteria, Chlamydiae, Vampirivibrionia, Micromonosporales, order Clostridiales, Bifidobacteriales, Erysipelotrichales, Micromonosporales, Vicinamibacterales, norank Clostridia, Chlamydiales, family Clostridiaceae, Bifidobacteriaceae, Oscillospiraceae, Erysipelotrichaceae, Micromonosporaceae, Lachnospiraceae, Polyangiaceae, Alcaligenaceae, genus *Clostridium sensu* stricto, *Lactobacillus*, *Bifidobacterium*, *Blautia*, UCG 005, *Agathobacter*, *Escherichia Shigella*, and *Pajaroellobacter* decreased significantly. This result suggests that the high starch diets changed the intestinal microbiota of largemouth bass, increasing the relative abundance of harmful bacteria (*Aeromonas*) and decreasing the relative abundance of beneficial bacteria (*Clostridium sensu* stricto, *Lactobacillus*, and *Bifidobacterium*) in the intestine.

### A High Starch Diet Induces Enteritis in Largemouth Bass

**FIGURE 4:**
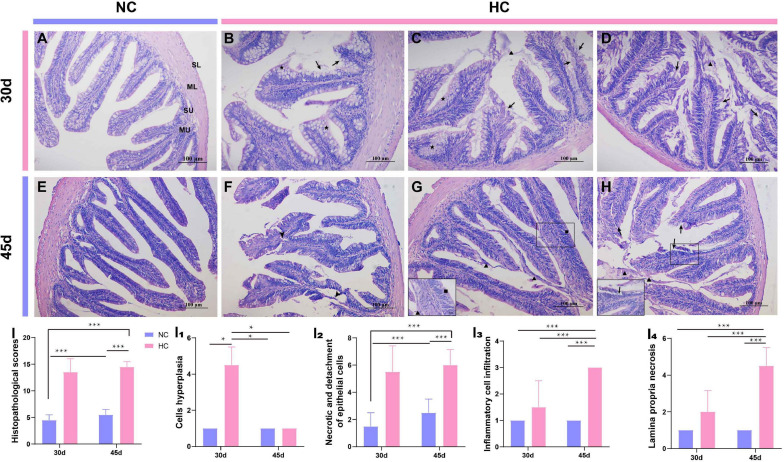
Histopathological observations of the largemouth bass intestine (H&E staining) **(A)** The intestines of the NC-fed fish were undamaged at 30 days, with an intact mucous layer (MU), submucosal layer (SU), muscle layer (ML), and serosa layer (SL); **(B–D)** Proliferation of mucus cells (pentagram), increased mucus secretion (triangles), and loss of epithelial cells were detected in the intestines of fish fed the HC diet for 30 days (arrow); **(E)** The intestines of NC-fed fish at 45 days; **(F–H)** the intestines of fish fed the HC diet for 45 days; intestinal villi broken (arrowhead), mucus secreted (triangles), and epithelial cells shed (arrow), respectively; **(I)** Total intestinal histopathological score; **(I_1_–I_4_)** the histopathological scores for cell hyperplasia, necrosis, and detachment of epithelial cells, inflammatory cell infiltration, and necrosis of the lamina propria, respectively (*P < 0.05, ***P < 0.001. NC: 0% α-starch diet, HC: 22% α-starch diet).

Numerous studies have shown that changes in the intestinal microbiota are closely related to the development of intestinal diseases (Sokol et al., 2006; Li et al., 2012). In this study, the high starch diets caused changes in the intestinal microbiota, but it was not known whether the intestine was diseased. Therefore, further observations of the changes in intestinal tissues of largemouth bass were used to determine whether intestinal diseases developed. H&E staining was used to investigate the changes in intestinal histology of largemouth bass. The results showed that after 30 and 45 days, fish fed the NC diet had a healthy intestine with an intact mucous layer (MU), submucosal layer (SU), muscle layer (ML), serosa layer (SL), and a smooth intestinal mucosa (Figures 4A,E); however, fish fed the HC diet developed significant damage to the intestine. A large proliferation of goblet cells in the intestinal mucosa of HC-fed fish, an increase in mucus secretion, a large amount of mucus adhering to the intestinal villi, damage to the intestinal mucosa, and necrosis and shedding of epithelial cells were observed after 30 days (Figures 4B–D). At 45 days, the proliferation of goblet cells decreased, but necrosis of the lamina propria, the intestinal villi ruptured, the layer of intestinal villi cells increased, and inflammatory cells infiltrated, while shedding epithelial cells and secreted mucus were still visible in the intestinal lumen (Figures 4F–H). According to the intestinal histopathological score, feeding the HC diet caused obvious damage to the largemouth bass intestine, which appeared to be moderate to severe necrotizing enteritis due to the increase in the number of goblet cells and mucus secretion and accompanied by shedding of necrotic epithelial cells and infiltration of inflammatory cells (Figures 4I–I_4_).

**FIGURE 5:**
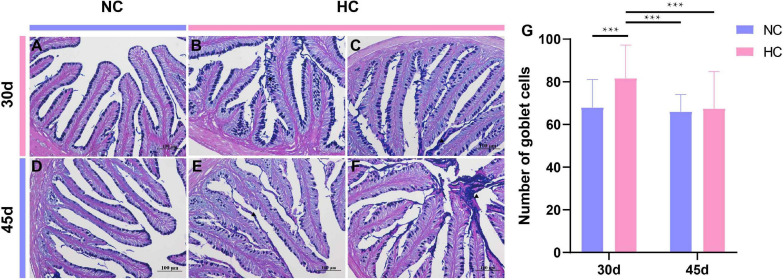
Intestinal goblet cells observations of the largemouth bass intestine (AB-PAS staining) **(A)** The number and distribution of intestinal goblet cells in fish fed the NC diet for 30 days; **(B,C)** proliferation of goblet cells (pentagram), increased mucus secretion (triangles) of fish fed the HC diet for 30 days; **(D)** number and distribution of intestinal goblet cells in NC-fed fish after 45 days; **(E,F)**, the number of goblet cells decreased in HC-fed fish at 45 days, and a large amount of mucus was secreted into the intestinal lumen (triangle); **(G)** number of intestinal goblet cells in largemouth bass fed the NC and HC diets for 30 and 45 days (***P < 0.001. NC: 0% α-starch diet, FC: 22% α-starch diet).

Alcian blue-periodic acid Schiff staining was used to further observe the changes in the intestinal goblet cells of largemouth bass fed the high starch diet. The results showed that the number of intestinal goblet cells was significantly higher in fish fed the HC diet than in fish fed the NC diet for 30 days (Figures 5A–C,G). The number of goblet cells was significantly lower at 45 days than at 30 days, and the number did not differ from that of fish fed the NC diet, but a large amount of mucus was visible in the intestinal lumen (Figures 5D–F,G). This may be related to the self-adaptive regulation by the organism.

**FIGURE 6:**
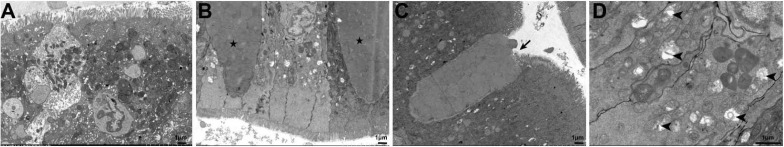
Ultrastructural pathology of the largemouth bass intestine after 45 days of feeding the two diets. **(A)** The intestinal cells of largemouth bass fed the NC diet were structurally intact and undamaged; **(B–D)** the intestinal cells of largemouth bass fed the HC diet. Mucoprotein-filled goblet cells (pentagram), goblet cells with enlarged apical openings (arrow), mitochondria with broken cristae, vacuolated, and unidentified membranous structures (arrowhead) were observed.

Organelle changes in the intestinal cells that occurred during the 45 days were further observed by transmission electron microscopy. The cell structure of fish fed the NC diet was intact and the striated border at the top of the cells was visible (Figure 6A), while the intestinal goblet cells of fish fed the HC diet were filled with mucoprotein (Figure 6B). Some goblet cells had enlarged apical openings (Figure 6C), and some intracellular mitochondrial cristae were broken or had disappeared, vacuolated, or contained unidentified membranous structures (Figure 6D). This was similar to the histopathological observations that a large amount of mucus was present in the intestinal lumen of fish fed the HC diet. These results suggest that a high starch diet could cause significant damage to the largemouth bass intestine.

### The High Starch Diets Alter Largemouth Bass Intestinal Digestive Function

**FIGURE 7:**
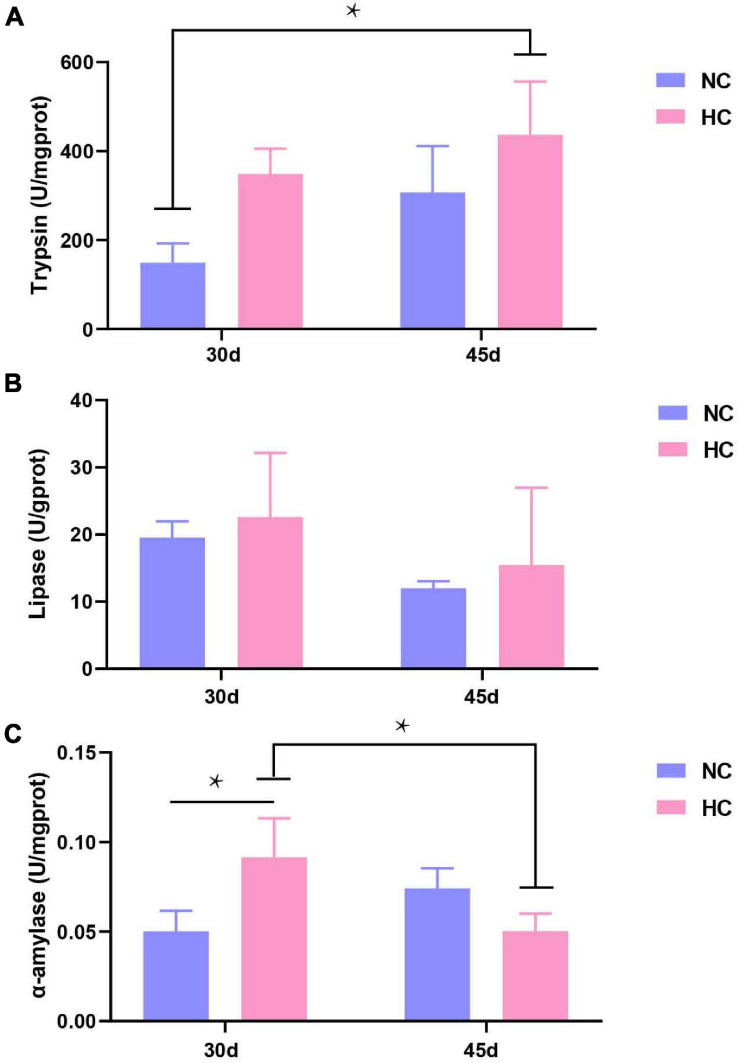
Intestinal digestive enzyme activities of largemouth bass **(A–C)** Trypsin, lipase, and α-amylase activity after 30 and 45 days, respectively (*P < 0.05. NC: 0% α-starch diet, FC: 22% α-starch diet).

The changes in the intestinal microbiota and the development of enteritis affect the digestive function of the intestine (Buts et al., 1999). Therefore, three common intestinal digestive enzymes were tested. As shown in Figure 5, no significant differences in trypsin or lipase activities were observed in either treatment group after 30 and 45 days (Figures 7A,B). However, the pattern of change in α-amylase activity was different. Amylase activity was significantly higher after 30 days in fish fed the HC diet than in fish fed the NC diet (*P* < 0.05), but the opposite trend was observed at 45 days. In addition, the α-amylase activity of the fish fed the HC diet for 30 days was significantly higher than that after 45 days (*P* < 0.05) (Figure 7C). This result suggests that largemouth bass adapt to a high starch diet by increasing intestinal amylase activity within 30 days, but if fed a high starch diet for a long time, they may not be able to effectively digest and absorb the starch.

## Discussion

The intestinal microbiota is an important component of the intestinal immune barrier and is closely related to various diseases, such as inflammation, non-alcoholic fatty liver, and obesity (Boulange et al., 2016). The intestinal microbiota is symbiotic in the intestine and can be affected by food (Flint et al., 2014). Numerous studies have found that a high-carbohydrate diet alters the intestinal microbiota. Feeding of juvenile rainbow trout (*Oncorhynchus mykiss*) with high carbohydrate induced intestinal microbiota disorders, with a significant increase in the relative abundance of *Aeromonas* sp., *Shewanella* sp., and γ*-*Proteobacteria (Geurden et al., 2014). Analysis of the intestinal microbiota of Chinese perch (*Siniperca chuatsi*) fed high starch showed a significant increase in the abundance of Tenericutes and a significant decrease in Fusobacteria (Zhang et al., 2020a). Largemouth bass fed a high starch diet developed disrupted intestinal microbiota with reduced abundance of intestinal probiotics (*Lactobacillus*) and increased abundance of potentially pathogenic intestinal bacteria (e.g., *Brevundimonas* and *Ralstonia*), which led to reduced intestinal acetate and butyrate concentrations, and impaired intestinal function (Zhou et al., 2020). In the present study, the high starch diet reduced the diversity and abundance of intestinal microbiota in largemouth bass, while the abundance of beneficial bacteria, such as *Clostridium*, *Lactobacillus*, and *Bifidobacterium* decreased. *Clostridium* is an important butyrate producing genus in the intestinal tract of animals (Vital et al., 2014). Butyrate is thought to improve the morphology of intestinal villus tissue, increase the activity of intestinal digestive enzymes, prevent intestinal diseases, and promote the growth of fish (Abdel-Latif et al., 2020). *Lactobacillus* and *Bifidobacterium* have been used as probiotics in fish, as they protect intestinal epithelial cells from damage and enhance disease resistance (Maji et al., 2016; Cavalcante et al., 2020; Ljubobratovic et al., 2020). Thus, the decrease in the abundance of *Clostridium*, *Lactobacillus*, and *Bifidobacterium* led to insufficient butyrate synthesis and probiotics and may induce enteritis in largemouth bass.

As one of the most important carbohydrates, starch is widely used in aquatic animal feeds, as it is considered one of the most inexpensive and readily available feed ingredients. However, most fish, particularly carnivorous fish, cannot efficiently use starch and chronic overconsumption leads to metabolic disorders and tissue damage or death (Tian et al., 2012; Wade et al., 2020). The activities of intestinal antioxidant enzymes, the length of the intestinal villi, and the thickness of the intestinal wrinkled were significantly reduced in *Oreochromis niloticus* fed a high carbohydrate diet (Xu, 2017). Ding et al. (2020) reported that intestinal villi of snakehead (*Channa argus*) became sparse and short as the carbohydrate content of the diet was increased (13–19%), and the intestinal villi were injured and shed at 19% carbohydrate content. In the present study, the intestines of largemouth bass developed moderate to severe necrotizing enteritis with an increase in the number of goblet cells and mucus secretion accompanied by shedding of necrotic epithelial cells and infiltration by inflammatory cells. These observations suggest that a high starch diet could damage the intestines of fish.

Trypsin, lipase, and α-amylase are the most important digestive enzymes in the intestine. α-Amylase metabolizes starch by breaking α-1,4-glycosidic bonds and facilitating digestion and absorption (Kim et al., 1999). In fish, α-amylase is mainly synthesized and secreted by the hepatopancreas (Ou, 2018) and is released by the intestinal mucosa to perform its physiological functions (Zhou, 2012). High protein and carbohydrate diets inhibit α-amylase activity (Xie et al., 2015; Hu et al., 2018). In the present study, long-term intake of the high starch diets resulted in reduced α-amylase activity. Largemouth bass that consumed high starch content developed damage to the hepatopancreas and insufficient α-amylase secretion. The changes in the intestinal microbiota affected the integrity of the intestinal mucosa, which affected the release of amylase and its attachment to the intestinal mucosa, leading to reduced α-amylase activity. However, more detailed interactions between the microbiota and the intestinal digestive enzymes need to be investigated in future studies.

## Conclusion

This study demonstrated that a high starch diet reduced the diversity and abundance of the intestinal microbiota in largemouth bass. The abundance of beneficial bacteria decreased, which may be associated with the development of enteritis. Our results illustrate the effects of a high starch diet on the intestines of largemouth bass. Intestinal health can be protected by adding probiotics to replace bacteria lost in this study (such as *Clostridium*, *Lactobacillus*, and *Bifidobacterium*). Therefore, our results provide a reference for conserving the intestinal microbiota of largemouth bass during modern intensive culture.

## Data Availability Statement

The datasets presented in this study can be found in online repositories. The names of the repository/repositories and accession number(s) can be found below: Sequence Read Archive (SRA) at the National Center for Biotechnology Information (NCBI) (accession number PRJNA730220).

## Ethics Statement

All animal handling procedures were approved by the Animal Care and Use Committee of Sichuan Agricultural University, following the guidelines of animal experiments of Sichuan Agricultural University, under permit number DY-2019202033.

## Author Contributions

XH, LZ, QK, and DC contributed to conception and design of the study and article writing and revision. LZ wrote the first draft of the manuscript. SL performed the statistical analysis. YF organized the database. YG, YO, SY, LY, and WL wrote sections of the manuscript. All authors contributed to manuscript revision, read, and approved the submitted version.

## Conflict of Interest

The authors declare that the research was conducted in the absence of any commercial or financial relationships that could be construed as a potential conflict of interest.

## Footnotes

1 http://drive5.com/uparse/

2 https://www.mothur.org/wiki/Download_mothur
