# Supplementary material for: A High Starch Diet Alters the Composition of the Intestinal Microbiota of Largemouth Bass Micropterus salmoides, Which May Be Associated With the Development of Enteritis
Source: Front Microbiol. 2021 Jul 8;12:696588. doi: 10.3389/fmicb.2021.696588 (PMC8297414; doi:10.3389/fmicb.2021.696588)
Supplement: Supplementary file 1 [file Table_1.DOCX]

***Supplementary Material***

Table S1. Valid sequence information for intestinal microbiota of largemouth bass fed with NC and HC.

| Amplified Region | Samples | Sequences | Bases(bp) | Average Length |
| --- | --- | --- | --- | --- |
| 338F-806R | 6 | 279431 | 116756431 | 417 |

Table S2. Alpha diversity index of intestinal microbiota of largemouth bass fed with NC and HC.

| Samples | Reads | |  |  | Community diversity | |  | Community richness | |
| --- | --- | --- | --- | --- | --- | --- | --- | --- | --- |
|  | Raw reads | Valid reads | Mean length |  | Shannon | Simpson |  | Ace | Chao |
| NC | 44196.00±4249.08 | 48360.67±15671.97 | 419.29±9.54 |  | 2.52±0.23 | 0.18±0.04 |  | 244.92±29.40 | 245.08±29.77 |
| HC | 48947.67±15689.63 | 43611.67±4217.98 | 418.46±0.10 |  | 1.93±1.33 | 0.41±0.41 |  | 202.53±42.78 | 203.24±43.41 |

Note: NC: 0% α-starch diet, FC: 22% α-starch diet


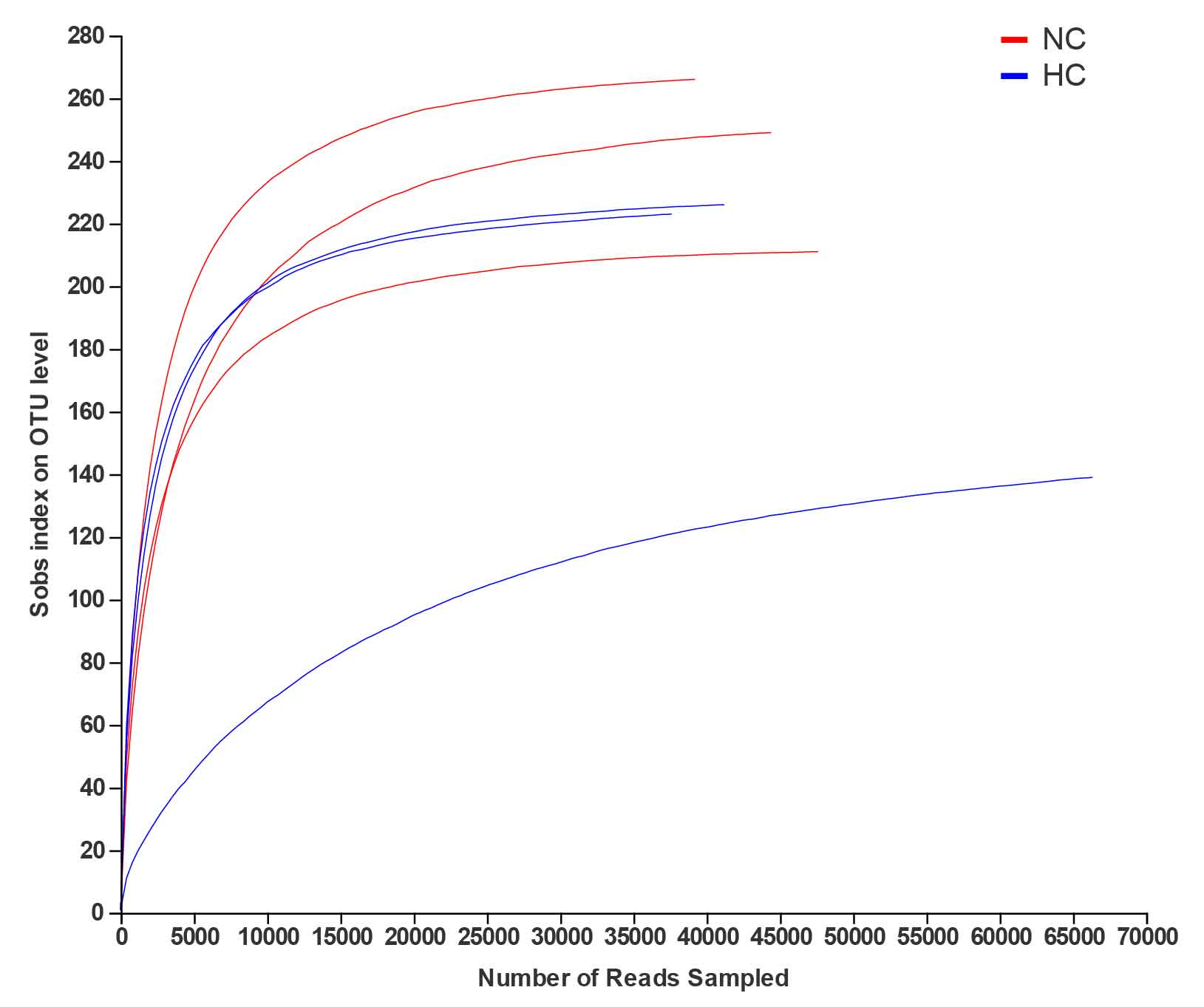


Fig.S1. Rarefaction curve of intestinal microbiota of largemouth bass

The rarefied curves for observed species number tended to approach the saturation plateau.
